# Investigation of complex miRNA-mRNA interaction networks during JAK inhibitor therapy in rheumatoid arthritis

**DOI:** 10.3389/fphar.2026.1904345

**Published:** 2026-08-14

**Authors:** János Rózsa, Dóra Csige, Monika Bodoki, Zsófia Hagymási-Szabó, Ferenc Tóth, Szilvia Szamosi, Ágnes Horváth, Nóra Bodnár, Edit Végh, Sándor Szántó, Gabriella Szűcs, Zsófia Pethő, Zsuzsanna Gyetkó, Levente Bodoki, János Kádas, Zoltán Szekanecz, Szilárd Póliska

**Affiliations:** 1 Genomic Medicine and Bioinformatics Core Facility, Department of Biochemistry and Molecular Biology, Faculty of Medicine, University of Debrecen, Debrecen, Hungary; 2 Doctoral School of Molecular Cell and Immune Biology, University of Debrecen, Debrecen, Hungary; 3 UD-GenoMed Medical Genomic Technologies Ltd., Debrecen, Hungary; 4 Department of Rheumatology, Faculty of Medicine, Institute of Internal Medicine, University of Debrecen, Debrecen, Hungary; 5 Department of Sports Medicine, Faculty of Medicine, University of Debrecen, Debrecen, Hungary

**Keywords:** gene expression, interaction analyses, JAK inhibitors, janus kinase, miRNA, response, rheumatoid arthritis, RNA-sequencing

## Abstract

**Background:**

MicroRNAs (miRNAs) are small molecules that contribute to the regulation of gene expression by binding to messenger RNAs (mRNAs), controlling the amount of proteins being produced. In the past few decades, biologic and targeted synthetic therapies (e.g., Janus kinase (JAK) inhibitors) have emerged for the treatment of rheumatoid arthritis (RA), which have promised new possibilities for treating the disease. By inhibiting the inflammatory cascade, these drugs are able to moderate further mRNA expression of different pro-inflammatory and inflammatory genes. Since none of the agents provide a uniform solution for all RA patients, a deeper investigation of gene expression processes and regulatory mechanisms, including miRNA-mRNA interaction networks, may facilitate a shift towards personalized medicine.

**Methods:**

In our current study, 28 active RA patients were recruited at the Department of Rheumatology, Debrecen, Hungary, blood samples were taken before enrolment and 6 months after continuous JAK inhibitor therapy (T0 and T6). After peripheral blood mononuclear cell isolation, total RNA was extracted, and next-generation sequencing (NGS) was performed to obtain miRNA expression pattern. Previously we examined the mRNA expression profile of the same patient group, using the results of this project, we conducted complex analyses to identify miRNA-mRNA interaction networks.

**Results:**

We analyzed the changes in miRNA expression patterns between T0 and T6 and identified 50 differentially expressed miRNAs. Response to treatment was determined by changes in DAS28 score. When comparing responder and non-responder groups, 33 and 30 miRNAs were differentially expressed at T0 and T6, respectively. Moreover miRNA-mRNA interaction network analyses revealed several regulatory mechanisms that may influence the pathogenesis of RA, such as cell proliferation, cytokine signaling, inflammatory response, and JAK/STAT signaling pathway.

**Conclusion:**

In all comparisons, several miRNAs were identified that may influence the pathogenesis of RA through the regulation of gene expression. According to responsiveness-related analyses the identified miRNAs were able to separate responder from non-responder at both T0 and T6, miRNA-mRNA interaction analyses identified hundreds of regulatory mechanisms which potentially affect the JAK inhibitor treatment and responsiveness to therapy. Determining the regulatory miRNAs of our previously identified mRNA biomarker candidates places our findings in a broader molecular content.

## 1 Introduction

Rheumatoid arthritis is one of the most common autoimmune diseases, affecting patients throughout their lives, influencing their quality of life and worsening their long-term health prospects (Szekanecz et al., 2007; Smith et al. 2011; Lee and Weinblatt, 2001). Inflammatory processes primarily have impact on small joints, but as the disease worsens, inflammation and tissue degradation can spread to bone tissue, and, in chronic cases, other organ systems (e.g., cardiovascular or respiratory systems) may also be affected (Lee and Weinblatt, 2001; Smolen et al., 2016; Szekanecz et al., 2011).

A prominent field of gene expression research in RA focuses on miRNAs, which are short, non-coding RNAs (ncRNA) consisting of 15–25 nucleotides that play a significant role in the posttranscriptional regulation of gene expression. During their expression, miRNAs undergo several maturation stages before the mature miRNAs become capable of fulfilling their regulatory functions, starting from the long primary miRNA (pri-miRNA) transcripts transcribed from DNA, which are then processed by Drosha nucleases to generate stem-loop precursor miRNA (pre-miRNA). In the next step, pre-miRNA fragments are cleaved by an enzyme (Dicer) into double-stranded miRNA, which is further modified into single-stranded and matured miRNA presented into the RNA-induced silencing complex (RISC). The mature miRNAs are able to bind at sequence specific manners to the target mRNAs, thus inhibiting their translation. Through the regulation of gene expression, miRNAs play a significant role in several biological processes, e.g., cell homeostasis, proliferation and differentiation, and in the regulation of innate and adaptive immune response (Ge et al., 2017; Feng et al., 2018; Ko et al., 2020; Vishnoi and Rani, 2023; Pepin and Gantier, 2016; van Wijnen et al., 2013). Numerous studies showed that changes in miRNA expression patterns may influence the pathomechanism of certain diseases, such as different types of tumors and autoimmune diseases like RA, systemic lupus erythematosus (SLE), systemic sclerosis (SSc) or dermatomyositis (DM). miRNAs also participate in the modulation of the molecular mechanisms of drug treatments (van Wijnen et al., 2013; Long et al., 2018; Zhao et al., 2026; Barbetta et al., 2025; Shi et al., 2024). In RA, several studies have already examined the roles of ncRNAs, including miRNAs, and found some miRNAs that may significantly contribute to the development of RA. Two novel examples are *hsa-miR-146a* and *hsa-miR-223*, which showed higher expression in RA patients compared to healthy controls and positively correlated with higher disease activity score (DAS28) (Wang et al., 2019; Liu et al., 2022; Taha et al., 2020). According to Yang et al. (2022), ncRNAs, including miRNAs, may therefore be key players in the diagnosis and treatment strategies of RA. Several miRNAs are reported to have role in the regulation of RA-related inflammatory and cell proliferation processes, such as *hsa-miR-34a*, *hsa-miR-106b*, *hsa-miR-27a-5p*, *hsa-let-7a-5p*, and *hsa-miR-23b* (Hou et al., 2019; Liu et al., 2020; Liu et al., 2019; Song et al., 2020; Vicente et al., 2016; Goto et al., 2021; Two et al., 2019; Zhao et al., 2025; Tang et al., 2022).

In the therapy of RA, there are different options from the conventional synthetic disease-modifying antirheumatic drugs (csDMARDs) through the biologics (bDMARDs) to the newest targeted, small-molecule inhibitors (tsDMARDs) (Singh, 2022; Szekanecz et al., 2024; Burmester and Pope, 2017; Rubbert-Roth et al., 2018). In the latter group we can find the JAK inhibitors, which primarily attack chemokine pathways or units binding to chemokine receptors, thereby targeting the silencing of signaling pathways, and ultimately the alleviation of inflammatory symptoms (Szekanecz and Koch, 2016; Kielbowski et al., 2024). The varying efficacy of bDMARDs and tsDMARDs in different patients, as well as the potential for side effects and allergic reactions, are significant issues that raise the need to identify potential biomarkers of response to different therapeutic agents (Singh, 2022; Szekanecz et al., 2024; Burmester and Pope, 2017; Rubbert-Roth et al., 2018; Szekanecz and Koch, 2016; Kielbowski et al., 2024). In their study, Lu et al. demonstrated that multi-omics analysis could reveal potential candidate biomarkers of response to tofacitinib treatment, which was the first approved oral JAK inhibitor in RA therapy (Lu et al., 2025).

In our previous study, we examined gene expression changes in active RA patients at the mRNA level in response to JAK inhibitor therapies. We examined the mRNA expression patterns of peripheral blood mononuclear cells (PBMC) of active RA patients who underwent JAK inhibitor therapy. We identified some differentially expressed genes (DEGs) between the responder (R) and the non-responder (NR) groups, which could be potential biomarkers of the responsiveness to JAK inhibitor therapies (Rozsa et al., 2025). In the current study, we aimed the completion of miRNA expression analyses of the same patient set and would like to present a more complex analysis to explore potential miRNA-mRNA relationships, which can provide deeper insight into gene expression changes and the potential posttranscriptional regulatory mechanisms, influencing the responsiveness and thus give a deeper understanding of the mechanism of action of JAK inhibitors in RA. Moreover, we would like to identify potential predictive miRNAs, which may also widen the spectrum and lead forward to personalized therapy in RA.

## 2 Methods

### 2.1 Patients

The patient population enrolled in the study was identical to the 28 patients (24 women, 4 men; age range: 33–71) recruited in our previous study. Before integration into the current research, the patients had to meet all inclusion and exclusion criteria.

The patients started baricitinib (10 women), tofacitinib (8 women and 4 men) or upadacitinib (7 women) therapies with possible supplementary treatments (MTX, medrol, NSAIDs) based on medical decision; one woman patient changed JAK inhibitor therapy from tofacitinib to baricitinib treatment. A patient was considered as responder to JAK inhibitor treatment if his/her DAS28 value fell below 3.2 after 6 months of continuous medication. All clinical data, inclusion and exclusion criteria and details regarding the specific JAK inhibitors and other supplementary treatments, are available in our published paper (Rozsa et al., 2025) and in Supplementary Table S1.

### 2.2 Peripheral blood mononuclear cell (PBMC) and total RNA isolation

PBMC fractions were separated from 10 mL venous blood samples (K_2_EDTA vacuum collection tubes) of the patients two times: before JAK inhibitor therapy (T0) and 6 months of continuous medical treatment (T6). Total RNA samples were subsequently isolated (Trizol-chloroform extraction) and then both concentration- (UV photometry) and quality-checked (capillary gel electrophoresis). The detailed methods for PBMC and total RNA isolation, as well as the RNA quality control procedures, have been previously described in published article (Rozsa et al., 2025).

### 2.3 Small RNA sequencing and data analysis

For small RNA-Seq library construction NEBNext Multiplex Small RNA Library Prep Kit (New England BioLabs, Ipswich, MA, USA) was used, according to manufacturer’s protocol. Briefly, a 3′ adapter ligation step was followed by RT primer hybridization and 5′ adapter ligation. Then cDNA was generated in reverse transcription reaction, and multiplex indexed primers for amplification of small RNS libraries. Libraries were then purified using QIAquick PCR Purification kit (Qiagen, Hilden, Germany), and AMPure XP magnetic beads (Beckman Coulter, Indianapolis, IN, United States) were used for size selection. Libraries were sequenced in single-end, 50bp sequencing run on Illumina NextSeq 2000 instrument (Illumina Inc., San Diego, CA, United States). An average 13.7 millions of raw reads were generated with Q30 > 90% base calling accuracy. StrandNGS 4.0 software (www.strand-ngs.com) was used for further data analyses. The aligner algorithm of the software with default settings was used, and raw reads were mapped to human reference genome version GRCh38, assembly GCA_000001405.15, while miRBase v22 annotation was used for annotating miRNAs. Approximately 94% of the reads were mapped and after the alignment step, the integrated DESeq algorithm of the software was used for quantification and normalization processes. Mann-Whitney paired and unpaired statistical tests without using fold change cutoff value have been performed to identify the differentially expressed miRNAs, p value <0.05 was considered statistically significant.

### 2.4 miRNA-mRNA network analyses

The miRBase database was used for the initial identification and annotation of miRNAs (Kozomara et al., 2019). To identify miRNA-mRNA interactions, TargetScanHuman 8.0 database was used since it represents a widely accepted gold standard in computational biology, leveraging a rigorously validated quantitative model. This tool incorporates seed-region evolutionary conservation, local AU content, and 3′UTR context to minimize false-positive rates. As a software upgrade, TargetScanHuman version 8.0 integrates advanced convolutional neural network (CNN) predictions based on comprehensive biochemical affinity measurements, significantly increasing the reliability of cell-level repression predictions compared to other algorithms (McGeary et al., 2019; Agarwal et al., 2015). The visualization of the results of potential interaction networks were generated in CytoScape 3.10.4 (Shannon et al., 2003).

## 3 Results

### 3.1 Changes in miRNA expression after 6 months of JAK inhibitor therapy (T6 versus T0)

**FIGURE 1:**
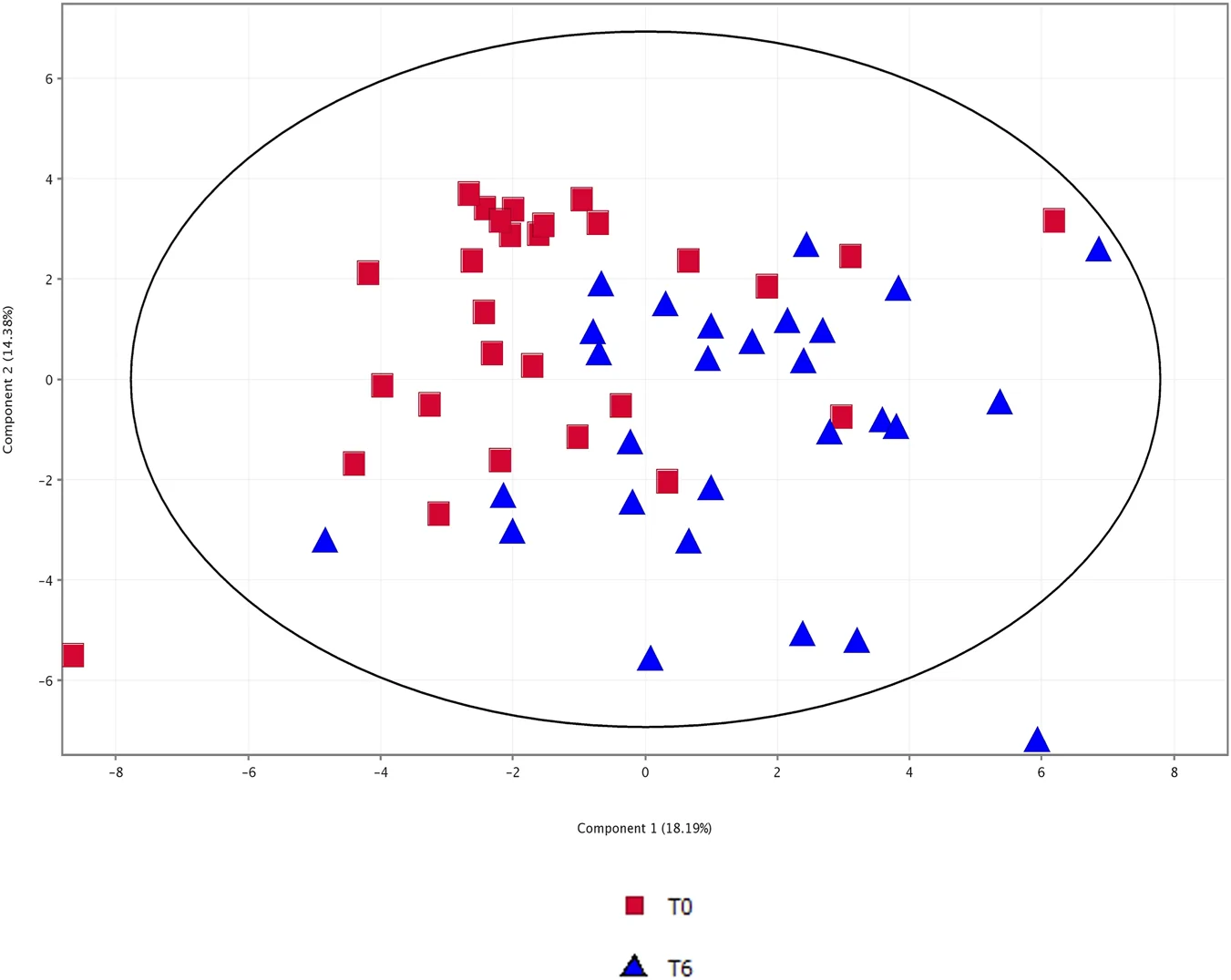
Principal component analysis of differentially expressed miRNAs between T6 and T0 samples. Red color represents T0 samples, blue color represents T6 samples.

As a first step, we examined how the 6 months of continuous JAK inhibitor therapy influenced the miRNA expression pattern, regardless of responsiveness. Our results showed that 50 miRNAs were differentially expressed between the two sampling time points, 28 were downregulated and 22 were upregulated at T6 compared to T0 (Supplementary Table S2). As for principal component analysis (PCA), it showed that these miRNAs were able to partially separate the samples from the two time points (Figure 1).

**FIGURE 2:**
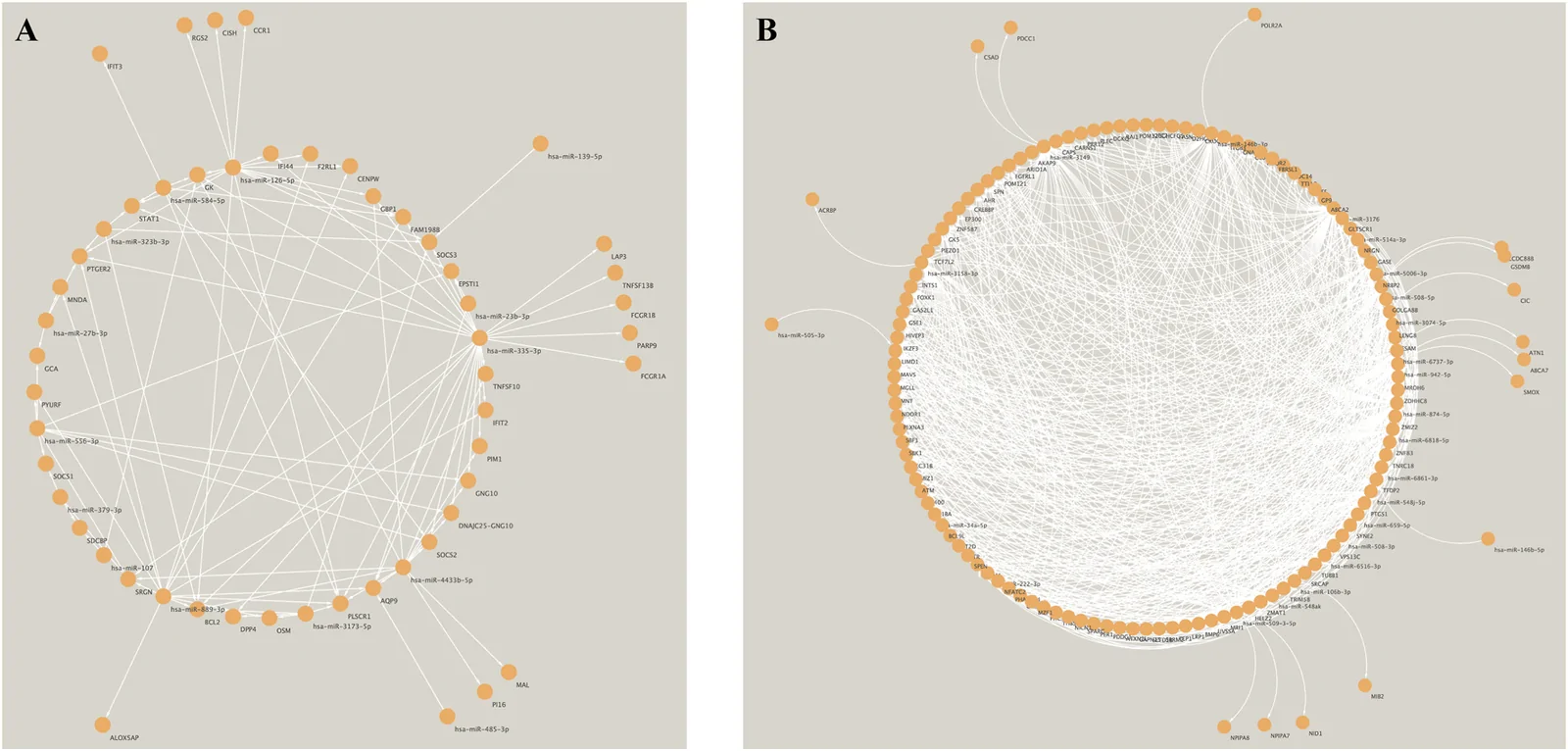
Interaction analysis of differentially expressed miRNAs and mRNAs between T6 and T0. **(A)** Interaction network between upregulated miRNAs and downregulated mRNAs. **(B)** Interaction network between downregulated miRNAs and upregulated mRNAs.

Using the TargetScanHuman 8.0 database, we predicted the potential interactions between the 50 differentially expressed miRNAs and the 225 differentially expressed mRNAs identified in our previous study (Rozsa et al., 2025). miRNA-mRNA regulatory interactions were considered to be relevant when the miRNA and its target mRNA showed opposite expression changes at T6, for example, upregulated miRNA and downregulated mRNA in T6 samples compared to T0. This analysis resulted in revealing 726 possible connections involving 150 differentially expressed mRNAs and 38 miRNAs (Supplementary Table S3, Figures 2A,B). Examining the miRNA hub nodes, 24 of the 38 miRNAs showed potential interactions with at least 10 mRNAs, 14 of them with at least 30 mRNAs. As a top posttranscriptional regulator, *hsa-miR-3176* proved to be the most interactive miRNA with 51 mRNA targets in this condition.

#### 3.2 Determination of differentially expressed miRNAs in terms of responsiveness

Following the classification method, 15 patients in the studied cohort were R and 13 patients were NR (Rozsa et al., 2025). Responsiveness-related miRNAs were examined separately at T0 and T6 sampling time points.

**FIGURE 3:**
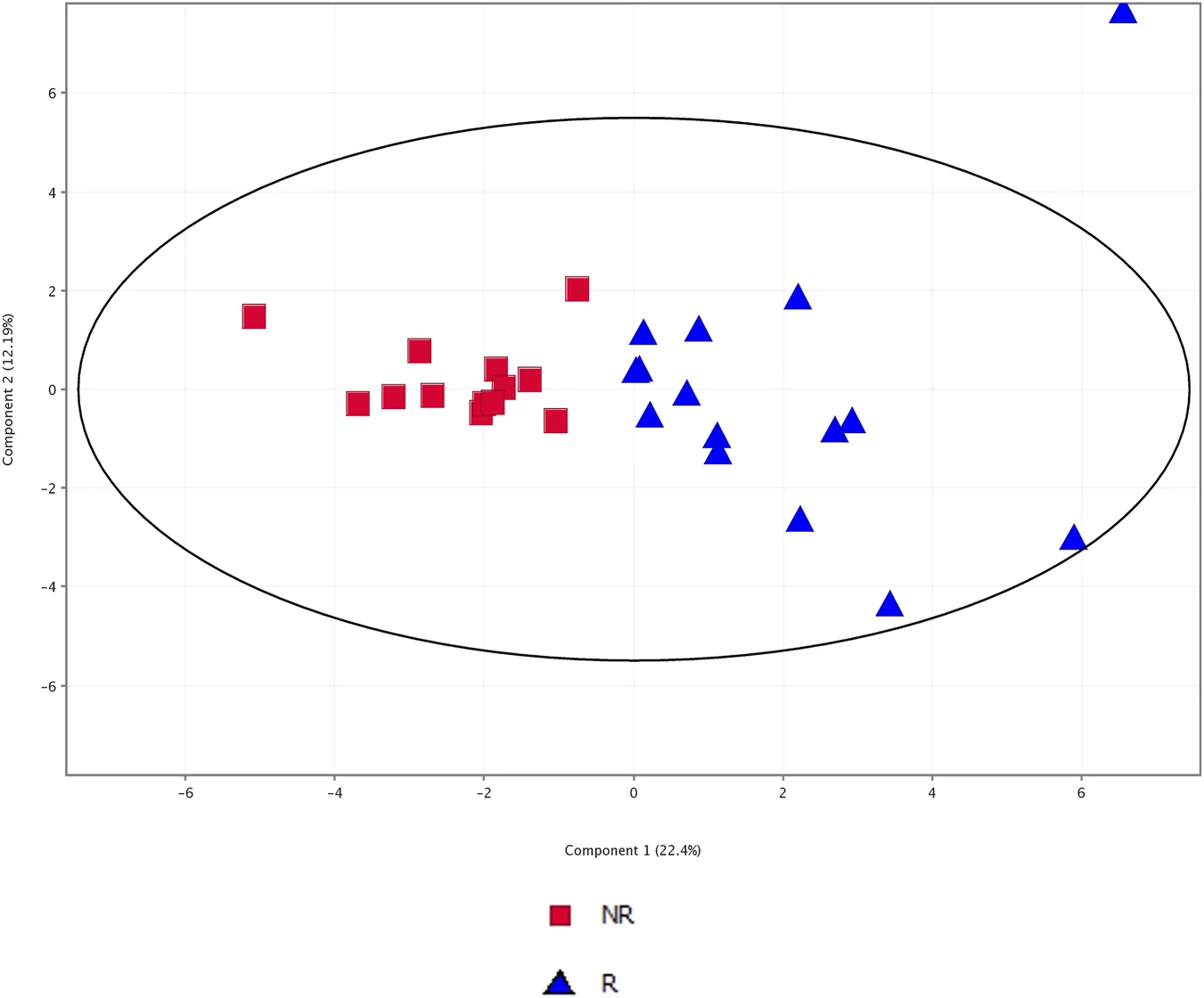
Principal component analysis of responsiveness-related, differentially expressed miRNAs at T0, demonstrating the relationship between the individual samples. Blue color represents responder (R) patients, red color represents non-responder (NR) patients.

**FIGURE 4:**
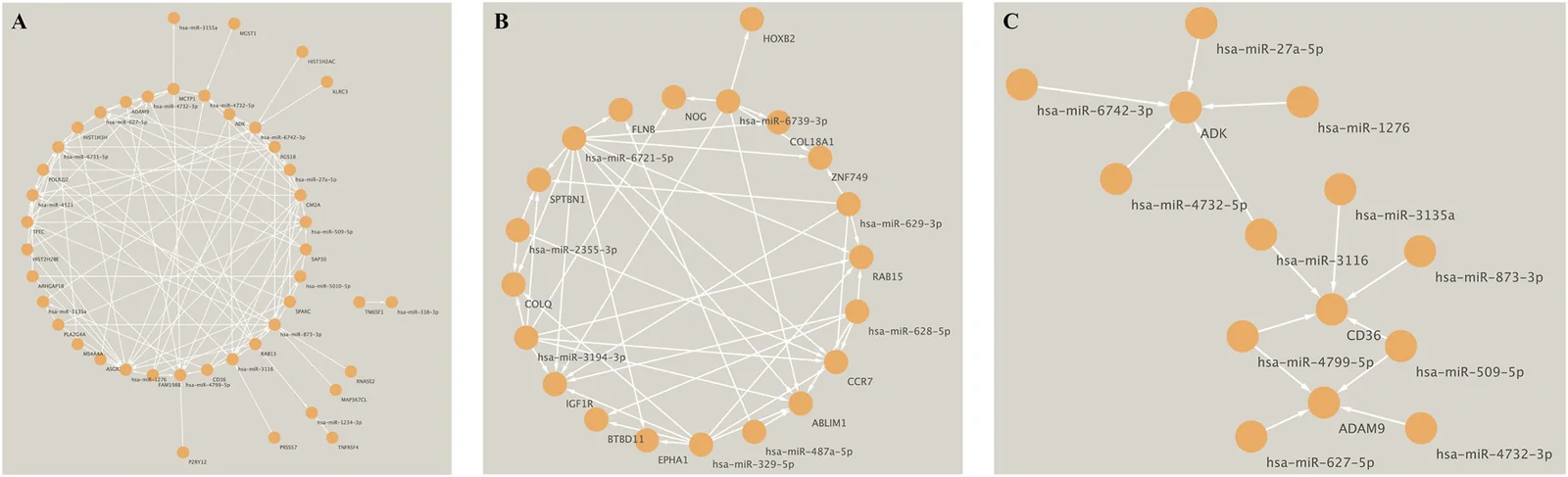
Interaction analysis of responsiveness-related, differentially expressed miRNAs and mRNAs at T0. **(A)** Interaction network between upregulated miRNAs and downregulated mRNAs. **(B)** Interaction network between downregulated miRNAs and upregulated mRNAs. **(C)** Interaction network between miRNAs and mRNA biomarker candidates.

At T0, 33 differentially expressed miRNAs have been identified, 10 down- and 23 upregulated (Supplementary Table S4). PCA demonstrated that this miRNA set is able to perfectly separate the R and NR patients based on their expression differences (Figure 3). The 60 responsiveness-related, differentially expressed mRNAs identified at T0 in our previous research and this set of miRNAs were used to find all possible miRNA-mRNA interactions (Rózsa et al., 2025), as a result of which 149 potential connections were revealed between 40 mRNAs and 25 miRNAs (Supplementary Table S5, Figures 4A,B). We investigated the main regulatory miRNAs and identified 3 of the 25 miRNAs that are able to interact with at least 10 possible targets, from which *hsa-miR-1276* showed to have the most (13) potential targets at T0. Furthermore, we uncovered 11 responsiveness-related miRNAs, which potentially modulate the posttranscriptional expression of three of our biomarker candidate mRNAs (*ADAM9*, *ADK*, *CD36*) from our previous project (Table 1, Figure 4C).

**TABLE 1:** Interaction analysis between responsiveness-relevant miRNAs and candidate biomarker mRNAs at T0.

mRNA	Regulation of mRNA	FC (gene)	Log FC (gene)	miRNA	Regulation of miRNA	FC (miRNA)	Log FC (miRNA)
*ADAM9*	Down	−1.301343	−0.38000122	*hsa-miR-4732-3p*	Up	1.645692	0.7186943
*ADAM9*	Down	−1.301343	−0.38000122	*hsa-miR-4799-5p*	Up	1.2591387	0.33243722
*ADAM9*	Down	−1.301343	−0.38000122	*hsa-miR-509-5p*	Up	1.5493728	0.6316843
*ADAM9*	Down	−1.301343	−0.38000122	*hsa-miR-627-5p*	Up	1.2565652	0.32948554
*ADK*	Down	−1.3776593	−0.46221918	*hsa-miR-1276*	Up	1.1324666	0.17946844
*ADK*	Down	−1.3776593	−0.46221918	*hsa-miR-27a-5p*	Up	1.4248368	0.5107966
*ADK*	Down	−1.3776593	−0.46221918	*hsa-miR-3116*	Up	1.5837756	0.663368
*ADK*	Down	−1.3776593	−0.46221918	*hsa-miR-4732-5p*	Up	1.4319295	0.5179604
*ADK*	Down	−1.3776593	−0.46221918	*hsa-miR-6742-3p*	Up	1.410502	0.49620867
*CD36*	Down	−1.45132	−0.5373657	*hsa-miR-3116*	Up	1.5837756	0.663368
*CD36*	Down	−1.45132	−0.5373657	*hsa-miR-3135a*	Up	1.6841884	0.7520535
*CD36*	Down	−1.45132	−0.5373657	*hsa-miR-4799-5p*	Up	1.2591387	0.33243722
*CD36*	Down	−1.45132	−0.5373657	*hsa-miR-509-5p*	Up	1.5493728	0.6316843
*CD36*	Down	−1.45132	−0.5373657	*hsa-miR-873-3p*	Up	2.183462	1.1266173

**FIGURE 5:**
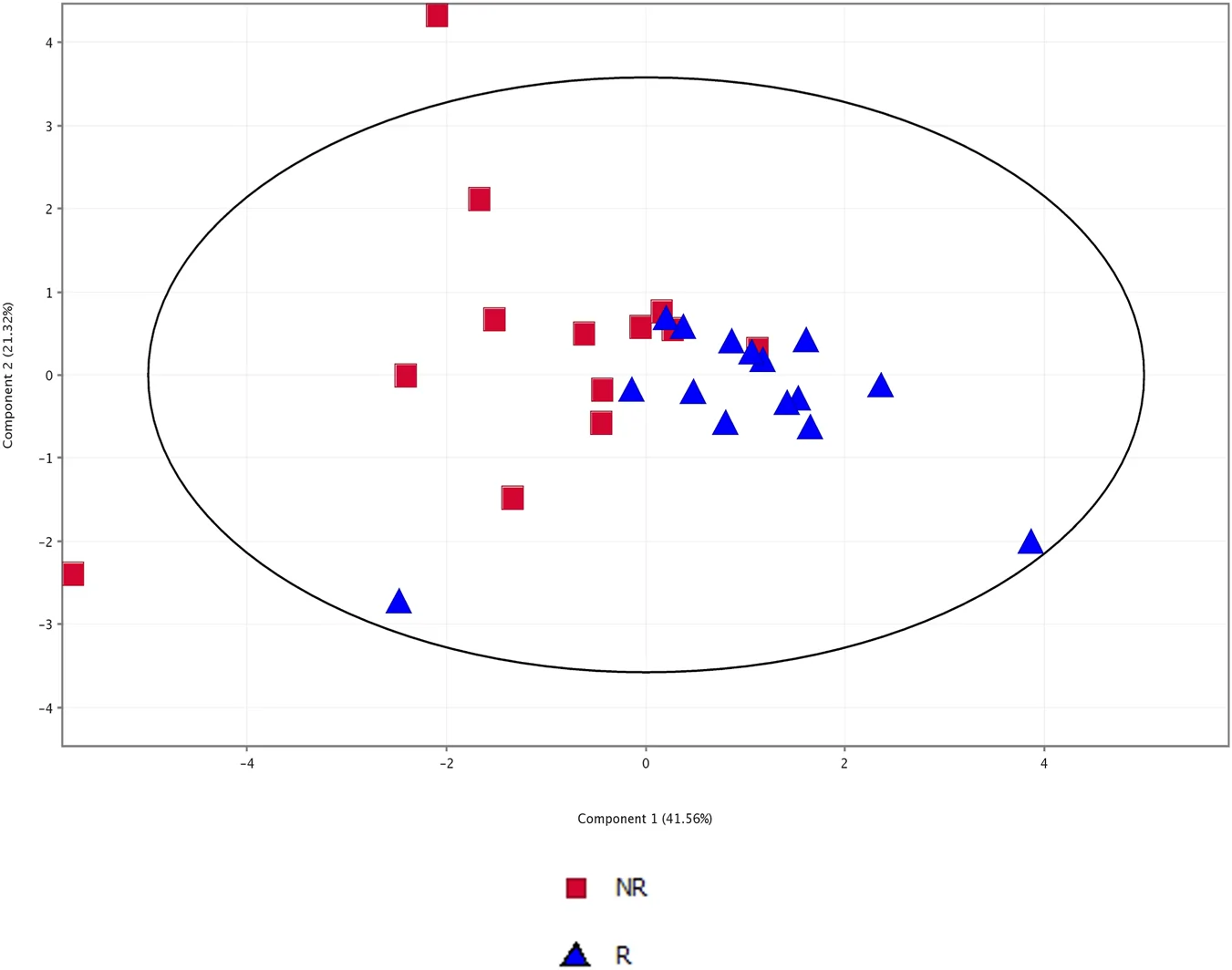
Principal component analysis of responsiveness-related, differentially expressed miRNAs at T6, demonstrating the relationship between the individual samples. Blue color represents responder (R) patients, red color represents non-responder (NR) patients.

**FIGURE 6:**
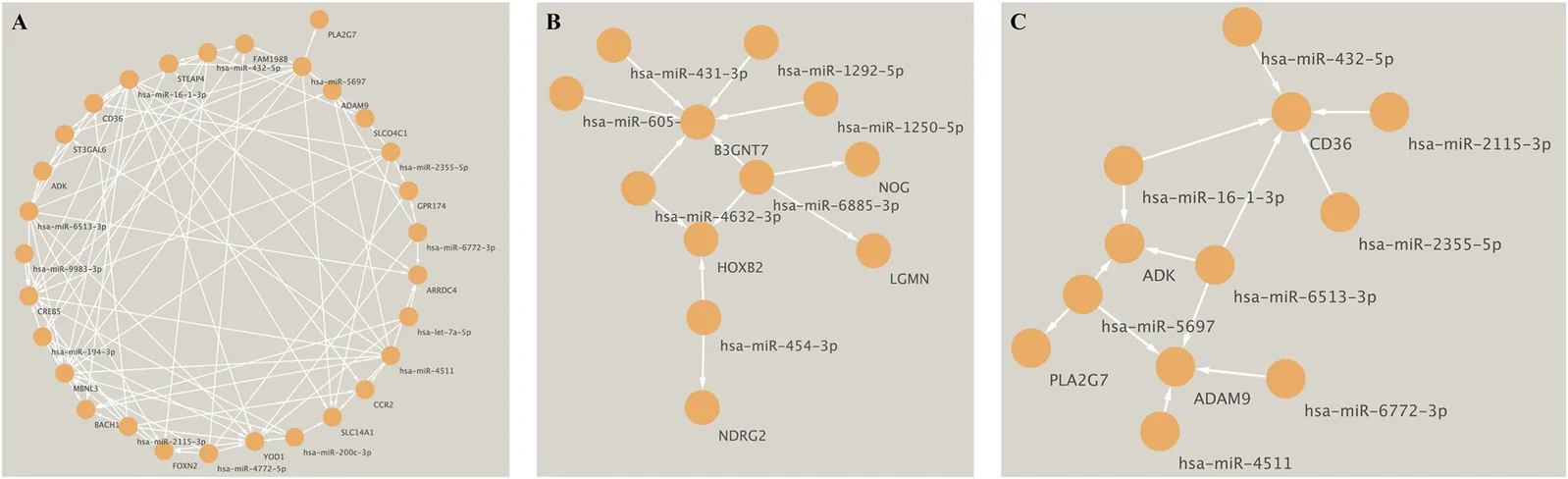
Interaction analysis of responsiveness-related, differentially expressed miRNAs and mRNAs at T6. **(A)** Interaction network between upregulated miRNAs and downregulated mRNAs. **(B)** Interaction network between downregulated miRNAs and upregulated mRNAs. **(C)** Interaction network between miRNAs and mRNA biomarker candidates.

Comparing R versus NR samples at T6, we were able to detect a total of 30 differentially expressed miRNAs, of which 11 were down- and 19 upregulated (Supplementary Table S6). These 30 miRNAs were able only to partially separate the R samples from NRs based on the PCA result (Figure 5). Only one common responsiveness-related miRNA has been identified between T0 and T6 sampling time points, *hsa-miR-9983-3p*. By examining the possible miRNA-mRNA interactions between the 66 differentially expressed mRNAs (Rózsa et al., 2025) and the 30 miRNAs, we revealed 97 connections involving 22 of the 66 mRNAs and 20 of 30 miRNAs, and only *hsa-miR-16-1-3p* and *hsa-miR-5697* are able to interact with 11 and 10 mRNA targets, respectively (Supplementary Table S7, Figures 6A,B). The expression levels of four of the five mRNA biomarker candidates (*ADAM9*, *ADK*, *CD36*, *PLA2G7*) are potentially regulated by eight responsiveness-related miRNAs. (Table 2, Figure 6C).

**TABLE 2:** Interaction analysis between responsiveness-relevant miRNAs and candidate biomarker mRNAs at T6.

mRNA	Regulation of mRNA	FC (gene)	Log FC (gene)	miRNA	Regulation of miRNA	FC (miRNA)	Log FC (miRNA)
*ADAM9*	Down	−1.3287206	−0.41003773	*hsa-miR-5697*	Up	1.1551957	0.20813729
*ADAM9*	Down	−1.3287206	−0.41003773	*hsa-miR-6513-3p*	Up	1.8478061	0.88581336
*ADAM9*	Down	−1.3287206	−0.41003773	*hsa-miR-4511*	Up	1.3211516	0.40179604
*ADAM9*	Down	−1.3287206	−0.41003773	*hsa-miR-6772-3p*	Up	1.7015207	0.76682466
*ADK*	Down	−1.3044841	−0.3834794	*hsa-miR-5697*	Up	1.1551957	0.20813729
*ADK*	Down	−1.3044841	−0.3834794	*hsa-miR-6513-3p*	Up	1.8478061	0.88581336
*ADK*	Down	−1.3044841	−0.3834794	*hsa-miR-16-1-3p*	Up	1.9921128	0.9942993
*CD36*	Down	−1.4290627	−0.51506925	*hsa-miR-2355-5p*	Up	1.7325509	0.7928977
*CD36*	Down	−1.4290627	−0.51506925	*hsa-miR-6513-3p*	Up	1.8478061	0.88581336
*CD36*	Down	−1.4290627	−0.51506925	*hsa-miR-2115-3p*	Up	1.4663804	0.5522594
*CD36*	Down	−1.4290627	−0.51506925	*hsa-miR-16-1-3p*	Up	1.9921128	0.9942993
*CD36*	Down	−1.4290627	−0.51506925	*hsa-miR-432-5p*	Up	1.7612197	0.81657493
*PLA2G7*	Down	−1.5977079	−0.67600363	*hsa-miR-5697*	Up	1.1551957	0.20813729

## 4 Discussion

The aim of our current project was to place our previous mRNA-level findings into a more complex context by analyzing the potential epigenetic regulatory mechanisms at the miRNA level. Our research also identified responsiveness-related miRNAs that may grow to be potential predictive biomarker candidates for JAK inhibitor therapies in RA after further research. The same samples of the same patients from our previously published study were examined. This cohort consists of 28 patients, 4 men and 24 women with active RA, and received JAK inhibitor therapies (10 patients underwent baricitinib treatment, 12 patients were given tofacitinib medication, and 7 patients started upadacitinib therapy).

First of all, we analyzed which miRNAs were differentially expressed after 6 months of continuous JAK inhibitor therapies compared to T0 samples, regardless of responsiveness. Our results showed significant expression differences in the case of 50 miRNAs, of which 22 were up- and 28 were downregulated. Two downregulated miRNAs, *hsa-miR-34a-5p* and *hsa-miR-106b*, have significant roles in the regulation of apoptotic processes in synovial fibroblasts (Hou et al., 2019; Song et al., 2020; Niederer et al., 2012), cell proliferation and the infiltration of inflammatory cells into the synovium in the affected joints (Liu et al., 2020). The upregulated *hsa-miR-335-3p* and *hsa-miR-335-5p* found to play a key role as inhibitors of osteoblast cell differentiation (Vicente et al., 2016), while *hsa-miR-23b* showed an elevated expression level in plasma samples of RA patients as compared to healthy controls (Liu et al., 2019; Vicente et al., 2016). *hsa-miR-126* may have dual influence in the pathogenesis of RA: through indirect regulation of the PI3K/AKT signaling pathway, it may be capable of inducing both cell proliferation and apoptotic processes in synovial fibroblasts (Gao et al., 2016), whilst its effects on DNA methylation processes in CD4^+^ T lymphocytes were examined as well (Yang et al., 2015). Focusing exclusively on the JAK/STAT signaling pathways, several of the 50 miRNAs have previously been proven to participate in the regulatory mechanisms. The downregulation of *hsa-miR-222* and *hsa-miR-34a*, and the upregulation of *hsa-miR-126* inhibits the expression of *JAK2* and *STAT3* (Cao et al., 2018; Xu et al., 2019; Khalife et al., 2020; Cheng et al., 2018), moreover, overexpression of *hsa-miR-34a* seems to induce Th1 and Th17 stimulation via major histocompatibility complex II presentation (Zhang et al., 2023) - our results correlate with these findings. The regulation of *hsa-miR-146b*, which has been studied in *STAT3*-based malignancies, has an important role in the crosstalk between NF-κB-dependent cytokine production (Xiang et al., 2014).

The interaction analysis in the condition of T6 versus T0 identified 726 possible interactions between 150 differentially expressed mRNAs and 38 miRNAs, and showed that these miRNAs may have posttranscriptional regulatory influence on numerous RA-relevant genes such as *STAT1*, *SOCS1*, *SOCS2*, *SOCS3*, *CISH*, *MDM4*, *TNFSF10*, which are related to regulation of inflammatory response, JAK/STAT and interferon-gamma-mediated signaling pathways (Yoshimura et al., 2005; Ma et al., 2021; Jin et al., 2024; Lu et al., 2026). 24 of the 38 miRNAs have potential interactions with at least 10 mRNA targets and 14 of which may be able to establish connection with at least 30 different mRNAs. Some of the 14 miRNAs, such as *hsa-miR-106b-3p*, *hsa-miR-126-5p* and *hsa-miR-335-3p* have been previously described to have potential pathogenic regulatory functions in osteoblasts and synovial fibroblasts. The top regulator based on our results is *hsa-miR-3176,* which showed 51 possible mRNA targets in the current condition.

After categorizing the patients into R and NR groups, we determined the responsiveness-related miRNAs at both sampling time points, identifying 33 (10 downregulated and 23 upregulated) and 30 (11 downregulated and 19 upregulated) miRNAs at T0 and T6, respectively.

At T0, *hsa-miR-27a-5p* showed higher expression level in R patients, this miRNA was reported to play significant roles in the regulation of JAK/STAT pathway, T-cell-mediated immune response, M2-like macrophage polarization, and it is considered to be a potential biomarker of tofacitinib treatment (Goto et al., 2021; Two et al., 2019; Zhao et al., 2025). Some other upregulated miRNAs were also found to regulate potential RA-related processes, such as *hsa-miR-338-3p*, which can reduce inflammatory processes via the PI3K/AKT and AMPK/Akt/mTOR signaling pathways in osteoarthritis (Hu et al., 2024), while elevated level of *hsa-miR-873* can enhance the Th17 cell differentiation in systemic lupus erythematosus (Liu et al., 2017). Interaction analysis revealed 149 potential miRNA-mRNA regulatory relationships involving 25 miRNAs and 40 mRNAs, including several RA-relevant genes, identified in our previous study, such as *CCR7 CD36*, *COL18A1*, *NOG*, *MS4A4A*, *PLA2G4A* and *SPARC* (Valdes and Spector, 2010; Hao et al., 2025; Ugarkar et al., 2003; Jiang et al., 2023; Amirzargar et al., 2020; Wierczeiko et al., 2024; Boutet et al., 2025; Moschovakis et al., 2019; Matsuno et al., 2002; Zhou et al., 2023). Regarding the regulation of JAK/STAT pathways, the upregulation of *hsa-miR-338-3p* directly inhibits the expression of *STAT1* (Liu et al., 2023), while *hsa-miR-1276* and *hsa-miR-299-5p* may interrupt the JAK2/STAT3 cascade (Zhang et al., 2024; Tian et al., 2023), and the role of *hsa-miR-27a-5p* has previously been described. During the investigation of the most potent regulatory miRNAs, only three miRNAs (*hsa-miR-873-3p*, *hsa-miR-1276*, *hsa-miR-6721-5p*) showed to have at least 10 mRNA targets (*hsa-miR-1276* showed the top number of potential interactions with 13 targets). Zhang and colleagues (2026) confirmed that *hsa-miR-1276* is significantly upregulated in PBMCs, and it may be a promising diagnostic marker–in this context it may enhance inflammatory processes, as opposed to its participation in the JAK/STAT pathway (Zhang et al., 2026). Moreover, 11 of responsiveness-related miRNAs may influence the mRNA level of *ADAM9*, *ADK* and *CD36*, which were proven to be potential predictive biomarkers for JAK inhibitor therapies in RA in our previous study (Rozsa et al., 2025). Three of the 11 miRNAs have shared target mRNAs; *hsa-miR-3116*, which is proven to have influence on the regulation of PI3K/AKT signaling pathway in glioma (Kong et al., 2020), can interact with *ADK* and *CD36*; *hsa-miR-4799-5p* and *hsa-miR-509-5p* can regulate *ADAM9* and *CD36*. Previous studies demonstrated that *hsa-miR-4799-5p* is related to hypertension and may participate in the pathomechanisms of Parkinson’s disease (Li et al., 2020; Zhang et al., 2021), while *hsa-miR-509-5p* is a potential predictive biomarker of survival in osteosarcoma (Guo et al., 2022). Placing emphasis on the most potent regulatory miRNA at T0, *hsa-miR-1276* may be able to interact with *ADK* from the validated responsiveness-related mRNAs.

Several of the responsiveness-related miRNAs at T6 were reported to have significant roles in different RA-related inflammatory processes. Such as the upregulated *hsa-miR-200c-3p*, which may inhibit integrin-mediated adhesion and infiltration of T cells into the site of inflammation (Mokmued et al., 2024), *hsa-let-7a-5p* has been identified as a potential diagnostic biomarker for RA (Tang et al., 2022), and overexpression of *hsa-miR-16-1-3p* was observed in PBMCs and synovial fluid, moreover, its levels correlated with clinical parameters (like DAS28 and CRP) (Jafri, 2023). Few of the downregulated miRNAs also participate in the pathomechanisms of RA, such as *hsa-miR-421* that intensifies inflammatory processes via fibroblast-like synoviocytes (Jiang et al., 2019), variants of *hsa-miR-431* were identified as risk and potential diagnostic biomarkers in RA influencing the NF-κB and JAK/STAT signaling pathways (Sadaty et al., 2025), while in the absence of *hsa-miR-454-3p*, an exacerbation of inflammatory processes has been observed in osteoarthritis (Ma et al., 2025). In addition, the previously mentioned miRNAs *hsa-let-7a-5p* (Yao et al., 2019) and *hsa-miR-200c-3p* inhibit the JAK2/STAT3 signaling pathway as well (Sajjadi-Dokht et al., 2022; Wu et al., 2022).

At T6 we identified 97 possible miRNA-mRNA interactions between 22 mRNAs and 20 miRNAs, which posttranscriptional regulatory mechanisms may have influence on such genes (*ADK*, *PLA2G7*, *NOG*, *CD36*, *BACH1*, *YOD1*, *CREB5*) that have previously been linked to the pathomechanism of RA in numerous studies (Zhou et al., 2023; Pelissier et al., 2025; Ma et al., 2024; Presti et al., 2025; Chien et al., 2020; Peter-Okaka and Boison, 2025; Xuan et al., 2025). The most special posttranscriptional regulator seems to be *hsa-miR-16-1-3p* with 11 potential targets. In addition, at T68 responsiveness-related miRNAs were proven to interact with targets like *ADAM9*, *ADK*, *CD36* and *PLA2G7*, which were identified as potential predictive biomarkers of responsiveness to JAK inhibitor therapies in our previously published study (Rozsa et al., 2025). There are overlaps between the regulated biomarker mRNA candidates; *hsa-miR-16-1-3p* may decrease the expression level of *ADK* and *CD36; hsa-miR-5697*, which was reported as biomarker in non-metastatic prostate cancer and SLE (Jafari Ghods et al., 2017; Jakobsen et al., 2016), can interact with *ADAM9, ADK* and *PLA2G7*; *hsa-miR-6513-3p*, which could be an exosomal biomarker for depressive symptoms in heart failure, can bind *ADAM9, ADK* and *CD36*. (Wang et al., 2024).

The investigation and identification of potential predictive biomarkers before the medical treatment, similarly to our finding in our previous mRNA-based study, would significantly contribute to the optimization of medical treatments for RA patients. By the integration of different biomarkers into fast-processed diagnostic tests, the samples of the patients may be screened before a therapy was chosen, as a result of which laboratory experiment and analysis the patients could receive the most appropriate medication in the fastest way possible to enable remission from inflammation. As another positive consequence, the healthcare system could benefit through smoother treatment strategies and economical savings as well.

The limitations of our study were the following: 1) the sample size was relatively small only with 28 patients, 15 R and 13 NR individuals were examined, and the generated data were not confirmed using independent technologies; 2) patient recruitment occurred at a single clinical center, and all recruited patients were white Caucasian, which may limit the generalizability of our results to broader patient populations; 3) our major goal was to identify all the miRNAs that show differential expression during the treatment and between R and NR groups, therefore statistical tests were applied without multiple testing correction; 4) we applied the widely accepted DAS28-based responsiveness categorization methodology without using stricter metrics such es CDAI, SDAI or Boolean Remission 2.0. 5) the results of miRNA-mRNA interaction analyses are premised on the TargetScanHuman v8.0 prediction-based database and none of the predicted interactions were confirmed experimentally. Beyond these limitations, we believe that our results could support the field of miRNA-based scientific research in JAK inhibitor therapies in RA.

## 5 Conclusion

In our current study, high throughput sequencing technology was used to obtain miRNA expression profile of patients with active RA undergoing JAK inhibitor therapies. Two sampling time points were analyzed, T0 (before starting the therapy) and T6 (after 6 months of continuous treatment). Comparing the T6 and T0 samples, we were able to identify several miRNAs which showed differential expression upon JAK inhibitor treatments, while after classification of our patient cohort based on their responsiveness to JAK inhibitors, several responsiveness-related miRNAs were revealed at both sampling time points. According to the literature, miRNAs in the three miRNA sets may have significant roles in the cell-level processes of various cell types involved in synovial inflammation (e.g., fibroblasts, osteoblasts), or have even been directly linked to the JAK/STAT signaling pathways and ultimately to the pathomechanism of RA. Using our previously published mRNA-Seq data set, which were generated from the samples of the same patient cohort, miRNA-mRNA interaction analyses were performed, and numerous potential regulatory mechanisms were identified, providing the opportunity to a more comprehensive understanding of the regulation of RA-related processes during JAK inhibitor therapies. Moreover, we could find potential regulatory miRNAs of our responsiveness-related mRNA biomarker candidates at both sampling time points.

We are concerned that further investigation of the identified miRNAs on larger patient cohorts and using independent methods, such as RT-qPCR, might confirm our results and their predictive biomarker potential for JAK inhibitor treatments, as a further step towards personalized medicine and more effective treatment strategies in RA.

## Data Availability

The datasets presented in this study can be found in online repositories. The names of the repository/repositories and accession number(s) can be found below: https://www.ncbi.nlm.nih.gov/, PRJNA1466767 https://www.ncbi.nlm.nih.gov/, PRJNA1322084.
